# Motivating Men Who Have Sex with Men to Get Tested for HIV through the Internet and Mobile Phones: A Qualitative Study

**DOI:** 10.1371/journal.pone.0054012

**Published:** 2013-01-08

**Authors:** Magaly M. Blas, Luis A. Menacho, Isaac E. Alva, Robinson Cabello, E. Roberto Orellana

**Affiliations:** 1 Epidemiology, STD and HIV Unit, School of Public Health and Administration, Universidad Peruana Cayetano Heredia, Lima, Peru; 2 Via Libre, Lima, Peru; 3 Epicentro, Lima, Peru; 4 Schools of Social Work and Community Health, Portland State University, Portland, Oregon, United States of America; Asociacion Civil Impacta Salud y Educacion, Peru

## Abstract

**Background:**

Men who have sex with men (MSM) have the highest HIV prevalence in Peru, yet they are underserved by traditional preventive programs. In Peru, the Internet and mobile phones have emerged as an effective and convenient tool to reach this population.

**Methods and Findings:**

From October 2010 to February 2011, we conducted eight focus groups with gay identified MSM (closeted and out-of-the-closet) and with self-identified heterosexual MSM in order to identify key features and preferences to be used to tailor culturally-appropriate messages that could be delivered through Internet and mobile phones to motivate MSM to get tested for HIV. Participants reported that in order to motivate HIV testing among MSM, interventions need to be based on motivational messages that encourage participants to overcome the fear of getting tested. Messages should increase the HIV risk perception (of participants who do not consider themselves at risk) by eliciting risky situations experienced by MSM. Messages should emphasize confidentiality, respect and the professionalism of the personnel conducting the counseling and testing. A thorough explanation of the process of HIV testing and the steps to follow after receiving the results should be provided. Messages should also contain information about the venue where the test will be conducted in terms of client characteristics, location, hours of operation and personnel. Finally, stigmatizing and stereotyping messages or images about “being gay” should not be included, as they act as deterrents for getting tested.

**Conclusions:**

Interventions aimed at motivating HIV testing among MSM should include motivational messages that reduce the fear of getting tested and increase the risk perception of participants. They should also market the venue where the testing will be conducted, the professionals who will perform the tests, and the type of tests available. Stigmatizing messages or images should be avoided.

## Introduction

In South America, most of the HIV epidemics are concentrated in and around networks of men who have sex with men (MSM) [1]. In order to decrease HIV transmission among MSM, innovative interventions to promote early identification and treatment of HIV and other sexually transmitted infections (STI) are urgently needed [2].

In Peru, sex between men represents the main route of HIV transmission [3]–[5]; the HIV prevalence among MSM in 2011 in the capital city of Lima was 12.4% [3]. A major barrier to HIV prevention efforts is a reported low frequency of HIV testing among MSM. Currently, in Lima the estimated percentage of MSM who have been tested for HIV and know their status is only 47.1% [3]. Alarmingly, it is estimated that around 50,000 individuals are still unaware of their HIV infection [6]. Some of the factors contributing to the low frequency of HIV testing include the absence of tailored health promotion strategies that address specific reasons and fears that MSM have for not getting tested for HIV, as well as the limited number of MSM who are reached by traditional interventions (mostly through peer educators) [2], [7].

Research conducted in Peru shows that the Internet and mobile phones are a promising medium to deliver HIV-related interventions. Peru has high Internet (26.2% by 2011) and cell-phone penetration (115% by March 2012), and MSM who surf the Internet have shown interest in receiving HIV prevention interventions through web pages, chat rooms, e-mails, e-groups and mobile phones [8]–[10]. Internet and mobile phones are also a good complement to existing peer education campaigns. In an online survey targeting MSM, only 11% of participants reported having been approached by a peer educator in the past year [7]. The Internet and mobile phones offer an innovative approach to target a broad number of MSM who are not being reached by traditional interventions.

The wide reach of the Internet also provides a timely vehicle to motivate MSM to get tested for HIV. Prior work demonstrated the efficacy of an online health promotion video-based intervention in increasing HIV testing among non-gay-identified MSM in Peru [2]. Another study found that free HIV testing can be effectively utilized as an incentive for participation in an online survey [10]. Mobile phones have also shown to be useful tools in supporting medication adherence and HIV transmission risk reduction among persons living with HIV in Lima. Additional research has shown that voice-recorded reminders and short message service (SMS) texting are acceptable ways to deliver behavioral change messages to this population [11].

Given the growing evidence of the Internet and mobile phones as prevention tools, an in-depth examination of the characteristics of messages that can influence the decision-making process of MSM who are ambivalent about getting tested for HIV may help improve web and mobile phone based interventions that aim at increasing HIV testing in Peru. The objective of this study is to identify key features and preferences to be used in the development of culturally-appropriate messages that could be delivered via the Internet and mobile phones to motivate MSM to get tested for HIV.

## Methods

### Focus groups

As part of an intervention study combining online video, e-mail, chat and mobile phone text messages aimed at increasing HIV testing among MSM in Lima, Peru, eight focus groups were conducted between October 2010 and February 2011. Three focus groups were conducted with self-reported, out-of-the-closet MSM, three with closeted gay-identified MSM; and two with MSM who self-identified as heterosexual. For the first two groups, we purposively recruited participants at a gay-serving community-based organization (Epicentro) and for the last group at billiard clubs, identified by previous research, as venues frequented by self-identified heterosexual MSM. Participants were assigned to the focus group oriented to gay-identified MSM if they self identified as gay. Participants who had not disclosed their gay identity to their family or friends were classified as closeted, and those who have disclosed their gay identity were classified as out-of-the-closet. Participants were assigned to the focus group oriented to non-gay-identified MSM if the participant self-identified as heterosexual or bisexual. Trans populations (transvestites, transsexual and transgender) were not included in this study because previous research in Peru showed that they have limited access to the Internet [2].

Focus groups were stratified by age. Among the focus groups conducted with out-of-the-closet MSM, two were with participants 18–24 years old and one with participants 25 and older. Among the focus groups conducted with closeted MSM, one was with participants 18–24 years old, and two were conducted with participants 25 years and older. Among the heterosexually-identified MSM, one focus group was conducted with participants 18–24 years old, and one with participants 25 years and older. Participants received an incentive of 10 Peruvian nuevos soles for transportation (about $4 US dollars) and a light meal for participating.

A focus group guide was developed based on previous research about the different reasons MSM have for not getting tested for HIV [7]. The content validity of the focus group guide was established with consultants who have worked in HIV prevention and control. At the beginning of the focus group participants were asked about their general knowledge about HIV and HIV testing. The initial discussion was followed by questions that elicited participant's preferences for how HIV-testing information should be delivered when using the following methods: email, mobile phones, chat and online videos. Examples of videos and email communications were shown to encourage discussions on what they liked, what they did not like, and how the key motivational messages could be improved in order to create a good product to encourage MSM to get tested for HIV. We showed a total of 6 videos of less than 10 minutes of duration with the following characteristics: 1) three dramas about gay participants getting tested, 2) one comedy, and 3) two video animations. We showed four types of emails with the following characteristics: 1) with formal language and statistical information about the HIV epidemic, 2) with informal language that included slang, 3) with images and little text, and 4) a standard email used by Epicentro to motivate HIV testing.

Participants were also asked about the advantages and disadvantages of receiving messages to motivate HIV testing through email, mobile phones, chat and online videos including their opinions as to the best times and frequency to receiving these messages via mobile phones and chat. The focus groups were conducted in Spanish by a trained moderator with experience in qualitative research. They lasted about one-and-a-half to two hours. The focus groups were digitally recorded and transcribed by the research staff. All transcriptions were compared with the audio recordings to assess their accuracy.

### Data analysis

Data were analyzed following a model adapted from Glaser and Strauss' grounded theory (1967) and Padgett's approach for content analysis (2008) [12], [13]. As the first set of transcripts became available, the research team met to review and identify emerging concepts and thematic categories. A set of codes based on these categories were identified independently by the researchers and used for subsequent transcript analyses. During the coding process, constant comparison was used to compare and review information given by participants from the different MSM groups and age categories (18–24 and 25 and older). This iterative approach was done in order to ensure the inclusion of the most appropriate categories and themes. After this, central messages or themes were inductively derived from the statements made by participants and key informants.

The investigators compared and discussed the results of their preliminary analyses until a consensus on the most important themes was reached. Later, we reviewed the transcripts to confirm our findings and identify quotes that best illustrated common themes. Quotes were translated and edited for ease of reading, but were not substantially altered. Our study was approved by the Institutional Review Boards (IRB) of the Universidad Peruana Cayetano Heredia and the non-governmental organization (NGO) Via Libre, both located in Lima, Peru. All participants gave verbal consent prior to their participation in the study. The IRB who reviewed this study waived the need for written informed consent from the participants.

## Results

### Demographics

We invited 64 individuals to participate in the study. A total of 60 MSM consented to participate, from which 58 (96.6%) actually completed the focus groups. Among the 58 participants, 20 were out-of-the-closet gay-identified MSM, 22 were closeted gay-identified MSM, and 16 were self-identified heterosexual MSM. The mean age of participants was 28 years.

### Participants' knowledge and perceptions about HIV and HIV testing

Participants reported that there has been wide dissemination of information regarding the prevention of HIV, and how important it is to the MSM community. One participant stated: *“…It is well known that it is an epidemic, a virus. I think we know a lot about this topic, is a topic that has always been important in the gay community.”* However, gay-identified MSM reported that in the general population there's still a lot of misinformation and the misperception that only gay men are affected by HIV.

Participants stated that they knew that *“HIV is now a chronic disease that is treatable; it is no longer a synonym of death.”* However, they also reported that there are still many people who think that HIV is a terminal disease. A participant said: *“In Lima, there are a lot of persons who think that having HIV is like you are going to die, that it is a terminal disease.”*

Participants also stated that HIV is a disease with high stigma and that there are several limitations for persons diagnosed with the virus. One participant stated: *“HIV is not a synonym of physical death but there are still high stigma and several social limitations for persons who live with HIV. For example heterosexual persons with HIV cannot get married, cannot have children.”*

### The negative influence of previous campaigns on HIV testing

Participants recognized that *“previous campaigns have focused mainly on condom use, but not on what happens if you dońt use a condom.”* They also reported that previous campaigns have been mainly oriented to heterosexual people rather than people with other sexual identities: *“The bad thing is that here they heterosexualize all the information. Here (in Lima) you have not seen the government making a spot for everyone.”*

Participants were asked to further discuss their experiences with previous HIV prevention campaigns. Most of the discussions centered on campaigns where getting infected with HIV was synonymous with death. A participant commented on one of these TV campaigns: *“I saw once a commercial where a boy was having sex with a girl, they took off their clothes and when the boy was on top of the girl, he put a gun over her head, the girl got scared and you see at the bottom of the screen a question like “Would you do this to the person you love? Protect yourself, protect your partner.”*

### Negative perceptions towards people who get tested for HIV

Focus group participants reported that there is a belief that if you get tested for HIV, people may think that you have several partners. As one participant commented: *“For example when I tell my friends that I had an ELISA, they get surprised, they look at me as if I were a sick person, promiscuous or something like that…that you are gay.”* The participant continued: *“If you tell them that you had an HIV test, they tell you…ah…you are homosexual, you probably have HIV.”* Another participant added: “…*the majority of persons think that if you have an ELISA it is because you are a source of infection*.”

### Strategies for Effective Message delivery for HIV Prevention

We identified 5 key themes with regards to preferences and features of an effective campaign to motivate HIV testing among Peruvian MSM: addressing fear, enhancing risk perception, explaining logistics, avoiding stigmatizing and stereotyped content, and using appropriate layout and language. These preferences were similar for participants 18–24 years old and for participants 25 and older. Results are presented by theme.

### 1. Addressing fear

Messages should encourage participants to overcome the fear of getting tested for HIV. Fear was cited as the main reason for not getting tested. According to participants, the main fear of testing positive is the *“possibility of dying”* or *“having your life turn upside down”.* Thus, messages should emphasize that whichever the result, the participant will be able to do the things he used to do. Messages suggested by participants include: *“Have fun, be happy, you will be able to do the things you used to do”* and, *“If you have it, is not a problem. Everything is treatable, but it is better to treat it on time. Trust us, it will be confidential.”*

Participants recommended that messages be phrased in a way that does not increase participants' fear of getting tested. Phrases such as *“if you turn out positive”* or *“if you need to start treatment”* should be avoided as they make people think they will be positive and they will have to start treatment. A participant stated: “*When I got tested the first time, I was very frightened about being positive, thus reading the phrase ‘if you turn out positive’ is not the idea that you want to have in your mind.”* It is more appropriate to provide the information in a subtle way or use the third person. For example, it is better to say *“if someone has risky sex…”* rather than *“if you had risky sex…”* or *“if a participant tests positive”* rather than *“if you test positive.”* Participants also recommended that in order to motivate HIV testing it is crucial to *“avoid showing or stating anything related to death due to HIV or complications related to the disease.”*

Participants agreed that in order to motivate HIV testing, interventions need to be based on *“motivational messages that transmit calmness.”* Participants mentioned that these messages should always be phrased in positive rather than negative tones. For example it is better to say *“It is better to know today”* rather than *“Dońt wait until is too late.”* Some motivational messages that participants suggested for a campaign include: *“Trust us, fun is not over”* and *“Erase your doubts, get tested today!”*

The messages should provide solutions for those with HIV positive test results. A participant stated “*They always raise the problem (getting infected with HIV), but not the solution, I have never seen an advertisement about HIV treatment.”* Also, participants commented that messages should emphasize that “*HIV is like other chronic diseases, such as diabetes or hypertension”* that “*the treatment now is free and will allow you to control the infection,*” that *“by testing earlier you may improve your quality of life and will reduce the probabilities of transmitting the virus to others,*” and that “*you will be able to continue your life normally and build a future.*”

### 2. Increasing risk perception

The messages should increase the risk perception of participants that do not consider themselves at risk by eliciting common risky situations, so that they can identify themselves with them. Examples that participants mentioned are: “*if you dońt know how you got home last night, get tested today! If you woke up and found someone you dońt know in your bed, get tested today!*” *“Have you ever forgotten to use a condom? Get tested!”* Stating messages as questions is helpful to prompt participants about risky situations they have experienced.

According to the participants, two concepts that should be included when sending these messages are 1) the concept of HIV spreading within social networks, because it reminds real and common situations about how HIV spreads (“*when you have sex with someone, you are having sex with all the network”)*, and 2) the concept of “HIV/AIDS does not show”, in order to remind people that a person can be HIV positive despite looking healthy. A participant stated: “*A person with whom you had sex could have looked normal and healthy but may have HIV.*”

### 3. Explaining logistics

We identified three core topics that participants need to know before deciding to get tested at a certain venue: the personnel who will be involved during the testing process, the place, and the process of HIV testing. This process should include information about the steps to get tested, the test itself, and the price of the testing. Below we explain these topics in detail.

### About the personnel who will do the testing

Messages should emphasize confidentiality, respect and the professionalism of the personnel who will do the testing. Participants reported that an effective campaign should have “*trained personnel that will respect you, that will keep your information private, that wońt make you feel ashamed and will treat you as a person.*” Also, the campaign should have “*personnel who you can trust,” and “who will make you feel relaxed and not alone.*”

Participants reported that the personnel who will do the counseling should be the same individual who will provide the results. One participant stated: “*The lower the number of persons who know your result, the better.*”

### About the place where the tests will be conducted

The messages should include detailed information about the venue where the test will be conducted in terms of clients, location, hours and personnel. Participants mentioned that *“it is important to include complete contact information: phone number, address, supportive institutions logotypes, names of key personnel, links to the project's website, and, when possible, use maps to show how to reach the health care facility*.”

The setting of the testing should be “*clean, friendly and cozy*” and should be pictured as a “*warm and pleasant place*” that “*participants can trust*” and “*that will make them feel relaxed.*” One of the fears of participants is to be identified as gay or HIV positive just for attending the venue where they will get tested, therefore it will be important to emphasize that not only gay or HIV positive people attend the venue. One participant stated: “*I have a lot of friends who want to come here (Epicentro) to get tested but they have fear of being recognized by someone they know.*”

### The process of HIV testing: during and after

The process of HIV testing should be clear to participants. Some important points that participants highlighted as important to know are 1) the level of pain they will feel, 2) the time they will spend getting tested, 3) the time to get the results back (participants preferred to receive the results the same day *“because it is very stressing to wait”*), 4) who will do the testing, 5) if the testing will be free and, 6) if other services are available. Emphasizing that the test is free, as well as the existence of other free services can encourage people to visit the center.

The messages should inform participants of all the steps they have to follow if they test positive for HIV. A participant said: “*I think that before you tell someone to get tested, you should explain what happens if a person tests positive for HIV.*” Also, it should be clear that if you test positive you will receive professional help to cope with the disease and to start your treatment. Another participant commented: “*If you test positive, they should refer you to someone who can explain to you how to start your treatment and continue with your life.”*

### 4. Avoiding stigmatizing and stereotyping content

The majority of participants emphasized that messages should not be perceived as stigmatizing. Gay-identified MSM (closeted and not-closeted) were very susceptible towards messages that implied that gay people have more sexual partners or are more promiscuous than other populations. Participants were also against messages that implied that gay people are more likely to have HIV because it generates fear among the population. One participant stated: “*If you say that MSM are the ones prone to get infected with HIV, you are generating fear.*” As a solution, one participant suggested the following message: “*The gay population is one of the populations that may acquire HIV because among all the types of sex, anal sex is the one with the highest risk.*” According to this participant, this message did not imply that gay men were promiscuous nor that they were more likely to have HIV; the message was centered on anal sex as the risk factor for acquiring the virus. Some symbols like the red ribbon were also perceived as stigmatizing because they are linked towards being gay and having HIV. One participant stated: “*I consider the red ribbon as a stigmatizing symbol. I see this ribbon in every message that wants to counsel people about HIV. Please do not use it. Imagine that the person you send it to is not out of the closet, someone can see the symbol and say that the person who received the message is a queer and has AIDS.*”

Both groups, gay-identified and heterosexually identified MSM, agreed that when using visual information such as pictures or videos, stereotyped caricatures of gay men should be avoided. One participant stated “*You should not use stereotyping stuff: not mannered (effeminate), not scandalous, not extravagant.*” The first group did not want these types of characters because they were perceived as stigmatizing and the second group because they did not identify with them. Also, neutrality is important for language. Scripts, emails, and text messages should contain neutral language and should avoid the use of the word HIV, AIDS, health center and gay-related jargon. A participant said “*Somebody else could read the (text) messages. Messages should use codes; you can create codes and avoid using health center or HIV because if someone reads it they may think that you are sick or have AIDS.*”

### 5. Using appropriate layout and language

Participants recommended that all text based information, when possible, should always be presented with images. One participant stated*: “When I have received information, it always has been leaflets with only text; unfortunately, people do not like to read. Information must include images.”* Other participants said: *“Images should include young and healthy people, friends or health care workers,*” “*images must show positive stuff*”, “*language must be simple and colloquial but not vulgar. They should be short and should change during the intervention; they should not be repetitive.”*

Regarding the layout, participants recommended avoiding the use of red, dark, single or gloomy colors, or colors related to HIV. A participant said: “*If you are inviting to HIV testing and use black color, what do you identify? Death!*” When possible, use humor because it makes information easier to understand and to remember. One participant stated: “*I think it is much better to use funny elements, because the problem when you want to talk about a serious topic is that they always use sensationalism, tragedy, crying, and soap-opera stuff. But when you use funny elements like a comedy, it attracts more attention…and incidentally the information gets into people's head.*”

## Discussion

We identified 5 key themes that should be considered when designing an effective campaign to motivate HIV testing: overcome fear to HIV testing, increase risk perception, explain logistics, avoid stigmatizing and stereotyping content, and use appropriate layout and language.

The first important component of an HIV testing campaign oriented to MSM is to provide messages to overcome the fear of getting tested. Previous studies have identified the “fear of the consequences of a positive test result” as the main barrier for not getting tested among MSM [7]. “Fear appeal” campaigns have been the basis of HIV prevention campaigns in Peru since the 1980′s. These campaigns have contributed to stigmatizing the disease and increasing peoples' fear of being infected with HIV and getting tested for the virus [14]. Future campaigns need to counteract this situation by providing motivational messages that transmit calmness and explain that HIV is now a chronic and treatable disease.

Although participants recommended that messages be phrased in a way that does not increase participant's fear of getting tested (avoiding phrases such as “if you turn out positive” or “if you need to start treatment”), they acknowledged the importance of receiving information about the steps a person has to follow if they test positive for HIV. Although these messages can seem contradictive, we believe they are not. The core message of an HIV testing campaign should not include phrases that can potentially increase participants fear. However they should include supplementary information about what happens if a person tests positive.

Messages should also focus on increasing risk perception. MSM with high-risk practices often do not perceive themselves at risk [15]. Thus, a brief explanation on the modes of transmission along with messages that can prompt participants to remember common risk situations they may have experienced would be useful to enhance risk perception [15].

A key aspect of a successful campaign includes the marketing of the personnel, the place, information about the process, including the test itself, and the price of testing. These components are congruent with elements of social marketing interventions which employ “marketing mix” strategies that include the product, price, place, and promotion [16]. These four elements are important to the planning and implementation of an integrated marketing strategy [16].

Following a social marketing research framework, in this case the product is represented by the benefits associated with HIV testing (e.g., early diagnosis of HIV infection). The price refers to the cost or sacrifice exchanged for the promised benefits. The price may carry intangible costs, such as embarrassment and psychological strain, as well as the monetary cost. The place refers to the location of the establishments where the HIV testing is performed; this includes the actual physical location, the operating hours, general attractiveness, comfort, and accessibility. Promotion includes “the type of persuasive communication we will use to convey product benefits and associated tangible costs and services, pricing strategies, and place components” [17].

Another important aspect to consider is the key social marketing principle of audience segmentation. Audience segmentation is based on the idea that audience members can be grouped into clusters according to characteristics that makes them homogenous in their response to a campaign or intervention [18]. In HIV prevention interventions oriented towards MSM, audience segmentation would be a must if the intervention needs to reach trans populations, as well as heterosexually identified MSM and gay identified MSM [2]. In our study, we only included gay-identified MSM (closeted and out-of-the closet) and heterosexually identified MSM from two age groups, and we did not find substantial differences that would make us think to stratify the intervention. One key element that emerged on all three sub-populations (even in the one that included out-of–the closet gay-identified MSM) was the importance of the inclusion of neutral characters (not stereotyped caricatures of gay men), as well as neutral language (absence of gay-related jargon) because either they were considered stigmatizing for the gay-identified group, or because they will not feel identified within the heterosexually-identified group.

Our study has some limitations. First, the results presented in this article are based on the comments of a purposively selected sample of MSM, therefore the study results should be used to inform health promotion strategies to motivate HIV testing, rather than direct guidelines for designing educational campaigns. Second, we recruited some participants at a community based organization that offers free HIV testing; therefore our sample is likely to be biased in terms of attitudes towards HIV testing. Additionally, we did not recruit trans populations. Given the sampling strategy, the generalizability of these results is limited. Despite the limitations our sampling strategy allowed us to reach saturation in our study as many of the responses were similar among participants.

In conclusion, the results of this study serve as the basis to design an effective campaign to motivate HIV testing among gay and non-gay identified MSM in Lima, Peru. These interventions should include motivational messages that reduce the fear of getting tested and increase the risk perception of participants. They should also market the venue where the testing will be conducted, the professionals who will perform the tests, and the test itself. Stigmatizing messages or images should be avoided.
